# Immuno-Metabolism: The Role of Cancer Niche in Immune Checkpoint Inhibitor Resistance

**DOI:** 10.3390/ijms22031258

**Published:** 2021-01-27

**Authors:** Chao-Yuan Weng, Cheng-Xiang Kao, Te-Sheng Chang, Yen-Hua Huang

**Affiliations:** 1 School of Pharmacy, Taipei Medical University, Taipei 11031, Taiwan; b313107058@tmu.edu.tw; 2 Department of Biochemistry and Molecular Cell Biology, School of Medicine, College of Medicine, Taipei Medical University, Taipei 11031, Taiwan; m120107009@tmu.edu.tw; 3 Graduate Institute of Medical Sciences, College of Medicine, Taipei Medical University, Taipei 11031, Taiwan; 4 School of Traditional Chinese Medicine, College of Medicine, Chang Gung University, Taoyuan 33382, Taiwan; 5 Division of Internal Medicine, Department of Gastroenterology and Hepatology, Chang Gung Memorial Hospital, Chiayi 61363, Taiwan; 6 TMU Research Center of Cell Therapy and Regeneration Medicine, Taipei Medical University, Taipei 11031, Taiwan; 7 International Ph.D. Program for Cell Therapy and Regeneration Medicine, College of Medicine, Taipei Medical University, Taipei 11031, Taiwan; 8 TMU Research Center of Cancer Translational Medicine, Taipei Medical University, Taipei 11031, Taiwan; 9 Center for Reproductive Medicine, Taipei Medical University Hospital, Taipei Medical University, Taipei 11031, Taiwan; 10 Comprehensive Cancer Center of Taipei Medical University, Taipei 11031, Taiwan; 11 PhD Program for Translational Medicine, College of Medical Science and Technology, Taipei Medical University, Taipei 11031, Taiwan

**Keywords:** metabolism, immuno-metabolism, metabolic reprogramming, immune checkpoint inhibitor, immunotherapy, resistance, tumor microenvironment, cancer niche

## Abstract

The use of immune checkpoint inhibitors (ICI) in treating cancer has revolutionized the approach to eradicate cancer cells by reactivating immune responses. However, only a subset of patients benefits from this treatment; the majority remains unresponsive or develops resistance to ICI therapy. Increasing evidence suggests that metabolic machinery in the tumor microenvironment (TME) plays a role in the development of ICI resistance. Within the TME, nutrients and oxygen are scarce, forcing immune cells to undergo metabolic reprogramming to adapt to harsh conditions. Cancer-induced metabolic deregulation in immune cells can attenuate their anti-cancer properties, but can also increase their immunosuppressive properties. Therefore, targeting metabolic pathways of immune cells in the TME may strengthen the efficacy of ICIs and prevent ICI resistance. In this review, we discuss the interactions of immune cells and metabolic alterations in the TME. We also discuss current therapies targeting cellular metabolism in combination with ICIs for the treatment of cancer, and provide possible mechanisms behind the cellular metabolic rewiring that may improve clinical outcomes.

## 1. Introduction

The discovery of checkpoint proteins has provided novel targets for cancer therapies, and the development of immune checkpoint inhibitors (ICI) has revolutionized clinical approaches to cancer. To date, there are seven food and drug administration (FDA)-approved ICIs for the treatment of different cancers [1]. Some patients with specific types of tumors have demonstrated durable responses from ICI treatment [2,3,4]; however, clinical outcomes for the majority of patients remain unsatisfactory. ICIs block co-inhibitory signals, such as cytotoxic T-lymphocyte-associated protein 4 (CTLA-4) and programmed cell death-1 and programmed death-ligand 1 (PD-1 and PD-L1) axis, to trigger immune responses and eradicate cancer cells [5]. Unfortunately, patients who received ICI treatment can develop resistance, which attenuates the efficacy of ICIs [6]. Faced with this clinical challenge, scientists are striving to understand the underlying mechanisms responsible for the development of resistance to ICI therapy. As more discoveries are reported, it appears that cellular metabolism plays a critical role in the development of ICI resistance [7,8,9]. In order to overcome resistance to ICI therapy, it is crucial to understand the metabolic features in cancer niches and the interrelationship between immune cells and cancer cells.

Inflammation, one type of cancer niche, is often associated with the proliferation and metastasis of cancer cells, leading to poor clinical outcomes [10]. Various interactions between cancer cells, stromal cells and immune cells can form an inflammatory tumor microenvironment (TME), which promotes cancer progression and metastasis [11]. Rapidly proliferating cancer cells can consume large amounts of oxygen, which decreases the oxygen availability and generates hypoxic regions [12]. With insufficient oxygen, immune cells may encounter environmental stresses, forcing them to undergo metabolic reprogramming [13,14]. In hypoxic regions, hypoxia-inducible factor (HIF) can become stabilized, and engage in the metabolic reprogramming of immune cells [15], which may further promote immunosuppression [15,16]. Cells with a high glycolytic rate generate vast amounts of lactic acid. Lactic acid has long been recognized as a waste product, however, it has recently been found to be an oncometabolite, and may be associated with the acidification of TMEs [17]. Several studies have found the acidity of TME to be an important factor in tumorigenesis and immunosuppression [17,18].

Because of the excessive use of nutrients by cancer cells, nutrients are depleted in the TME, leading to harsh conditions that induce immune cells to alter their metabolism of glucose, amino acids and lipids to adapt to the nutrient-restricted conditions [19]. During this cancer-induced metabolic reprogramming, some immune cells differentiate or polarize into immunosuppressive phenotypes [8], while other immune cells lose their anti-tumor functions [20,21]. Together, the dysfunctional metabolisms can impede immune responses to cancer cells, and also create an immunosuppressive TME that allows cancer cells to escape from immune surveillance.

Cellular metabolism has recently been found to be an important factor in developing ICI resistance, and is regarded as a clinical barrier for ICI treatment [22]. In order to overcome this clinical challenge, several ongoing clinical trials are targeting cellular metabolic pathways in combination with ICIs to yield better clinical outcomes.

## 2. Cancer Niches

### 2.1. Inflammation

**Figure 1:**
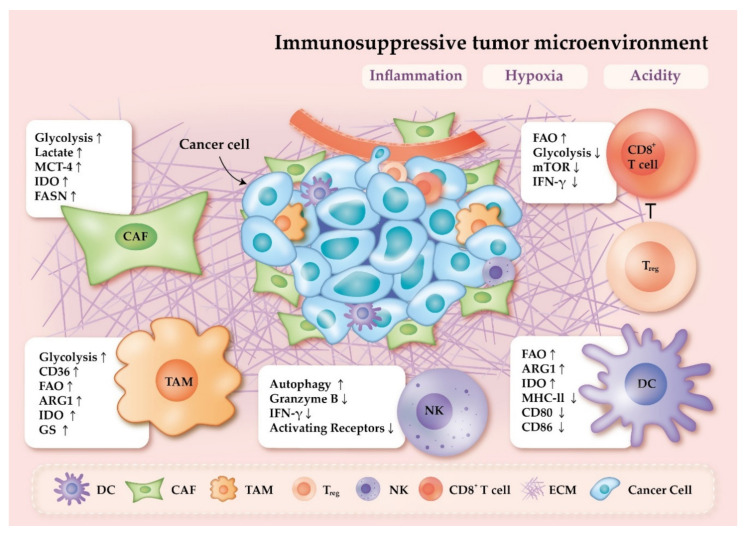
Immune cells undergo metabolic reprogramming within the immunosuppressive tumor microenvironment (TME). Inflammation, hypoxia, and acidity are the three hallmarks in the TME, resulting in immunosuppression, cancer progression, and metastasis. Cancer associated fibroblasts (CAFs) are key players in generation and regulation of extracellular matrix (ECM). Stiffened ECM can promote glycolysis in CAFs and support cancer cells. Excessive production of lactate by CAFs can be transported via monocarboxylate transporter-4 (MCT-4) and leads to the acidification of the microenvironment. The expression of arginase 1 (ARG1) and indoleamine 2,3-dioxygenase (IDO) in tumor associated macrophages (TAMs) can contribute to the inhibition of effector T cells. TAMs can also accumulate lipid via scavenger receptor CD36 and serves as a source of fatty acid oxidation (FAO) used for differentiation and tumor promotion. Similar to TAMs, ARG1 and IDO expression are also upregulated in dendritic cells (DCs), which leads DCs toward a more immunosuppressive state. The decreased expression of major histocompatibility complex class II (MHC-II) could also impede antigen presentation by DCs and attenuate T cell-mediated immune responses in hypoxic conditions. The elevated activity and number in regulatory T cells (T_regs_) may impede CD8^+^ T cells effector functions. Anti-cancer immunity property of CD8^+^ T cells and natural killer (NK) cells are attenuated by the dysregulation of metabolism in the TME. FASN, fatty acid synthase; mTOR, mammalian target of rapamycin; GS, glutamine synthetase; IFN-γ, interferon-gamma.

Inflammation is a hallmark of cancer and is associated with the growth and progression of cancer cells. Cancer cells, peripheral stromal and inflammatory cells can together form an inflammatory TME, which in turn promotes proliferation, progression and metastasis of cancer cells [11]. It has been reported that inflammatory cytokines such as interleukin-6 (IL-6) and tumor necrosis factor alpha (TNF-α) in the TME are able to enhance PD-L1 expression in cancer cells [23,24], contributing to escape from T cell immune surveillance. Tumor-associated macrophages (TAM) are abundant in the TME, and may take part in regulating inflammation. It has been shown that inflammatory cytokines IL-23 produced by TAMs can induce inflammation and promote tumor growth and progression in a colorectal cancer mouse model [25]. Additionally, macrophage-derived TNF-α can augment the PD-L1 expression in cancer cells [24], thus impeding T cells anti-tumor functions. The presence of IL-6 in the TME enhances the glycolysis activities in cancer associated fibroblasts (CAF), enabling them to generate metabolic intermediates to support cancer cells [26,27]. Similarly, IL-6 also induces glycolytic enzyme expression in cancer cells via signal transducers and activators of transcription 3 (STAT3)/c-Myc signaling [28], and correlated with tumorigenesis, which together suggests that inflammation can induce metabolic reprogramming and promote cancer progression (Figure 1).

### 2.2. Hypoxia

Tumor cells consume large quantities of oxygen due to their high rate of proliferation; this leads to the formation of hypoxic regions. Hypoxia is a hallmark of TME and a common characteristic of solid tumors [29], where tumor cells proliferate rapidly to build up solid tumor masses, and accompanied with abnormal formation of blood vessels that may not function properly to supply oxygen into the tumor masses [30]. Tumor cells can adapt to these hypoxic regions by activating HIF-1 transcription factor, enabling tumor cells to shift their metabolic profile from oxidative phosphorylation (OXPHOS) to glycolysis [30]. HIF-1, a crucial factor in regulating angiogenic factors such as vascular endothelial growth factor (VEGF) and vascular endothelial growth factor receptor (VEGFR), is overexpressed in several cancers and correlated with metastasis and poor prognosis [31]. The expression of HIF-2α is significantly enhanced in several cancers [32,33,34], and can increase cancer stem-like properties by activating Wnt and Notch pathways and stem cell related markers such as c-Myc, octamer-binding transcription factor 4 (Oct4) and Nanog [35]. Interestingly, HIF-2α is largely expressed and regulates Oct4 in embryonic development stage, which controls division, differentiation and function of stem cells [36]. In line with this, the HIF-2/Oct4 axis has been identified in regulating stemness of embryonic germ stem cells [37,38]. Additionally, HIF-2α can promote hypoxic cancer cells to proliferate via boosting c-Myc transcriptional activity and cell-cycle progression, and together promote proliferation and tumorigenesis [39]. Moreover, HIF-2α has also been reported to elevate the expression Oct4 and Sox2, contributing to stemness and invasiveness characteristics [40].

Under hypoxic conditions, the decreased rate of proteolytic degradation causes HIF-1α to accumulate. Elevated expression of HIF-1α in hypoxic regions may impair the ability of natural killer (NK) cells to upregulate surface activating receptors such as NKp46, NKp30, NKp44, and NKG2D [41]. Depletion of HIF-1α decreases the cytotoxicity in NK cells, but significantly delays tumor growth via stimulating non-productive angiogenesis [42]. The augmented production of adenosine through ectonucleotidases CD39 and CD73 in hypoxic region impedes NK cells cytotoxic activity and cytokine production [43,44], and activation of adenosine A_2A_ receptor (A_2A_R) in NK cells suppress their maturation and proliferation in the TME [45]. Additionally, cancer cells can activate autophagy under hypoxia, which degrades NK-derived granzyme B, thereby impeding NK-mediated tumor lysis [46,47].

In hypoxic regions, TAMs are shaped into tumor promoting phenotype, and upregulate platelet-derived growth factor (PDGF) and VEGF to support the growth of cancer cells [48]. It has been reported that TAMs reside in hypoxic tumor regions and significantly upregulate the expression of regulated in development and DNA damage responses 1 (REDD1), which inhibits mammalian target of rapamycin (mTOR) activity [13]. The inhibited mTOR may further impede glycolysis in TAMs, leading to the formation of abnormal blood vessels and facilitating metastasis [13]. TAMs may also facilitate tumor hypoxia by competing for available oxygen in the TME, impeding T cell infiltration [49].

Limited oxygen in hypoxic regions may lower expression levels of major histocompatibility complex II (MHC-II), CD80 and CD86, as well as levels of proinflammatory cytokines such as IL-1β, IL-6, and TNF-α in dendritic cells (DC), thus impairing the maturation and functions of DCs [50]. Additionally, differentiation of DCs under hypoxic conditions may impede their antigen uptake and alter their chemokine expression [51], which may further affect DCs ability for triggering immune responses.

A recent study has reported that hypoxia in the TME induces T cell mitochondria dysfunction, which decreases the production of ATP and mitochondrial OXPHOS activities, leading to T cell exhaustion [14]. T cell exhaustion may be associated with the downregulation of mitochondrial fusion protein mitofusin 1 and upregulation of miR-24 [14]. Additionally, the ability to proliferate and to produce interferon-gamma (IFN-γ) is attenuated in CD8^+^ T cells under hypoxic conditions [52]. Reintroduction of oxygen can restore the cytokine producing capacity of T cells [52]. It has been reported that under the hypoxic region, HIF-2α downregulates Fas-ligand expression and induce the expression of A_2A_R in natural killer T cells, leading to immunosuppression [53]. In addition, studies in a colitis-associated colon cancer mouse model have demonstrated that hypoxic conditions reduce differentiation of CD4^+^ effector T cells, while elevating the number and activity of regulatory T cells (T_regs_) [54]. These findings suggest that a hypoxic TME may have detrimental effects on effector T cells, and may attenuate anti-tumor responses (Figure 1).

### 2.3. Acidity

Increased production of lactic acid by cancer cells can cause acidification of the TME since lactate and H^+^ are transported outward by monocarboxylate transporter-4 (MCT-4) [55]. The decreased pH in acidic TMEs promotes tumor growth and metastasis [56]. Recently, lactate has been suggested to be a key player in cancer; it has been associated with the development of malignancies, immune escape, and regulation of cytokine release [57] (Figure 1).

It has been shown that when co-cultured with MCF7 breast cancer cells, CAFs are induced to express MCT-4 [58]. The increased glycolysis in CAFs may produce excessive lactate that is transported via MCT-4 leading to acidification of the TME.

Interestingly, a report by Colegio et al., sheds some light on the effect of lactic acid on TAMs. They show that cancer-derived lactic acid induces the expression of VEGF in TAMs, and skews TAMs toward a M2-like phenotype [59]. They also demonstrate that lactate upregulates the expression of arginase 1 (ARG1) in TAMs [59]. ARG1 plays a critical role in tumor promotion, as the ARG1-dependent pathway is responsible for generating cell proliferating substrates. It was also shown that extracellular acidosis can promote macrophage polarization toward a tumor-promoting phenotype in a prostate cancer model [60]. Neutralizing the tumor-secreted acids could reduce the pro-tumor phenotype of TAMs and impede tumor progression [60]. Interestingly, low extracellular pH may also be involved in regulating inflammatory cytokines and phagocytic activity in monocytes and macrophages [61].

Lactate, an oncometabolite in the TME, is a robust regulator of T cells [55]. It has been reported that CAF-derived lactate decreases the population of anti-tumoral CD4^+^ T cells, while increasing the population of T_regs_ in a prostate cancer model [62]. This reduction in anti-tumoral CD4^+^ T cells might result from lactate dependent SIRT1-mediated T-bet deacetylation [62]. On the other hand, lactate might enhance the activity of NF-kB and Foxp3 expression, which induces naïve T cells to polarize into T_regs_ and creates an immunosuppressive TME to sustain cancer progression [62]. Additionally, a recent study demonstrates that accumulation of lactate can upregulate the expression of lactate transporter SLC5A12 in CD4^+^ T cells, increasing IL-17 production and fatty acid synthesis (FAS) and reducing glycolysis [63]. Another study revealed that, an acidic environment can significantly impede secretion of IFN-γ and TNF-α by T cells, preventing them from generating proinflammatory cytokines [64]. The inhibition of glycolysis is also observed in acidic conditions, abrogating the activation of T cells [64]. Administration of bicarbonate can neutralize tumor acidity, increasing T cell infiltration and enhancing the efficacy of immunotherapy [64].

Acidity and lactate in the TME have also been demonstrated to impede the cytotoxic functions of NK cells. A recent report indicates that the activities of T cells and NK cells are impaired via inhibition of nuclear factor of activated T cells (NFAT) mediated by lactate dehydrogenase A (LDHA)-associated lactic acid production and intracellular acidification. The inhibited NFAT in T cells and NK cells restricts their IFN-γ production [65], abrogating their anti-tumor responses. Likewise, tumor-derived lactic acid causes intracellular acidification in liver-resident NK cells, leading to dysfunction of mitochondria, and inducing apoptosis of NK cells in biopsies from colorectal liver metastasis patients [66].

### 2.4. Cancer Associated Fibroblasts (CAF)

CAFs are especially abundant in solid tumors, and have various functions in the TME, such as promoting cancer growth and metastasis, as well as regulating the extracellular matrix (ECM) [67]. CAFs can directly promote tumor growth by secreting stromal cell-derived factor 1 (SDF-1), which mediates the recruitment of endothelial progenitor cells, thereby promoting angiogenesis [68]. Additionally, CAFs play a role in the assembly of fibronectin, which is involved in metastasis, and express other major ECM components that promote tumor progression [69].

### 2.5. Extracellular Matrix (ECM)

ECM is a non-cellular component produced by the secretion of intracellular resident cells that provides both biochemical and structural support. Alterations towards both degradation and stiffness of ECM can promote tumor growth and progression [70]. In solid tumors, tissue containing high amounts of ECM proteins may stiffen the stroma, thus promoting further malignancy [71]. It was recently reported that stiffened ECM may enhance glycolysis and glutamine metabolism in both cancer cells and CAFs, demonstrating the metabolic interplay between these cell types [72]. Aspartate secreted by CAFs is utilized by cancer cells for nucleotide biosynthesis to maintain proliferation. While glutamate secreted by cancer cells is utilized by CAFs in balancing redox state through the glutathione pathway to remodel ECM [72].

### 2.6. Nutrients and Immune Cells Metabolic Reprogramming

Rapidly proliferating cancer cells have high biosynthetic demands and generate nutrient-deficient TME [73]. The scarcity of nutrients, including glucose, amino acids and fatty acids, may induce immune cells to undergo metabolic reprogramming that can affect their fate and functions [73,74] (Figure 1).

#### 2.6.1. Glucose Metabolism

Glucose is rapidly consumed by cancer cells, leading to low levels in the TME, and a glucose-deficient TME decreases the anti-cancer immunity property of CD8^+^ T cells [20]. The metabolism of glucose is distinct in different subsets of T cells [75]. Naïve T cells circulate throughout the body looking for antigen, requiring a small amount of glucose to generate ATP via the tricarboxylic acid (TCA) cycle and OXPHOS in order to maintain their functions [75]. mTOR appears to play central roles in regulating the quiescence state of naïve T cells and in their activation [76]. mTORC1 induces transcription of Myc, while mTORC2 increases the expression of glucose transporter 1 (GLUT1), which enhances glucose uptake [76]. During the activation of naïve T cells to activated T cells, there is a metabolic switch from OXPHOS to aerobic glycolysis [75,76,77]. Massive consumption of glucose by cancer cells limits the availability of glucose in the TME, dampening mTOR activity, glycolytic activity and IFN-γ production in T cells [20]. This glucose-deprived TME, however, may be beneficial for developing T_regs_, since they mainly fuel their energy needs through OXPHOS, and only require a small amount of glucose [78,79]. Under the stress of nutrient deprivation, autophagy activity is upregulated in order to maintain survival [80]. Interestingly, a recent study demonstrated that autophagy related gene Atg5 suppresses the expression of GLUT1, which impedes glucose metabolism and the production of IFN-γ in CD8^+^ T cells [81].

Glucose is an essential fuel for cellular growth and cytokine production in NK cells. Glucose is utilized through glycolysis and OXPHOS in activated NK cells, and is further metabolized via the citrate–malate shuttle [82]. This distinct metabolic pathway is mediated by the transcription factor Srebp, and also takes part in the production of IFN-γ and granzyme B in NK cells [82]. Interestingly, the expression of fructose-1,6-bisphosphatase (FBP1) is elevated in NK cells during lung cancer progression. The increased FBP1 expression attenuates NK cell cytotoxicity and cytokine production by inhibiting glycolysis [21]. As such, inhibition of glucose metabolism in NK cells may impede their effector functions, thus promoting cancer growth and progression.

CAFs are the predominate type of stromal cells in the TME, and utilize aerobic glycolysis to generate nutrients that fuel cancer cells [83]. The reliance of CAFs on aerobic glycolysis may be driven by oxygen availability in the tumor site, HIF-1α stabilization, transforming growth factor-β (TGF-β), or PDGF signaling [84]. It has been shown that TGF-β- or PDGF-stimulated CAFs can undergo a shift from OXPHOS to aerobic glycolysis via downregulation of isocitrate dehydrogenase 3α, which stabilizes HIF-1α protein to promote glycolysis [85]. Interestingly, exosomes secreted by CAFs inhibit mitochondrial OXPHOS in cancer cells, while enhancing glycolysis and glutamine-dependent reductive carboxylation [86]. CAF-derived exosomes may also contain metabolites such as amino acids, lipids, and TCA cycle intermediates, and can be harnessed by cancer cells under nutrient-deprived conditions [86].

It has been reported that the metabolic profile of macrophages can be altered during polarization. While M1-like macrophages display characteristics of high glycolytic activity, increased uptake of glucose drives macrophages toward a proinflammatory phenotype [87], M2-like macrophages depend on OXPHOS [87], and exhibit immunosuppressive properties. M2-like TAMs are metabolically distinct from M2 macrophages, and utilize glycolysis [88], while also secreting TNF-α to promote glycolysis in cancer cells [49], thus facilitating tumor growth.

Interestingly, most cancer cells upregulate glycolysis, and downregulate OXPHOS. However, researchers have recently discovered that OXPHOS is upregulated in several kinds of cancers, such as leukemias, lymphomas, and pancreatic ductal adenocarcinoma [89], and the elevated OXPHOS may promote cancer metastasis and progression [90].

#### 2.6.2. Amino Acid Metabolism

Tryptophan, an essential amino acid in humans, plays a key role in regulating the function of immune cells and may be metabolized into kynurenine by the enzyme indoleamine 2,3-dioxygenase (IDO), which is often associated with immunosuppressive properties [91,92,93]. Myeloid-derived suppressor cells (MDSC) isolated from breast cancer tissues display elevated IDO expression and are correlated with increased infiltration of T_regs_ [94]. The enhanced IDO-expressing MDSCs as well as T_regs_ suppress T cell anti-tumor activity. It has been found that ectopic IDO increases M2 macrophage related markers such as IL-10 and CXC chemokine receptor 4 (CXCR4), and decreases CCR7 and IL-12p35 M1 related markers [95]. It has also been reported that hypoxic hepatoma cells induce IDO expression in macrophages, thereby suppressing T cell proliferation and promoting the expansion of T_regs_ [96]. Additionally, elevated expression of IDO in DCs stimulated by IFN-γ or TGF-β can lead to T cell suppression [97,98]. Similarly, augmented IDO expression in CAFs is observed in human esophageal cancers [99], and CAFs can also recruit and convert DCs into IDO-producing regulatory DCs [100], depleting tryptophan bioavailability, which consequently impedes effector T cell activation and proliferation [99,100]. Interestingly, it has been reported that higher levels of kynurenine, a tryptophan metabolite, are found in different types of cancers compared with normal tissues [101], and correlate with the suppression of T cell proliferation [102]. Kynurenine, may also inhibit NK cell proliferation and cytotoxicity and inducing NK cells apoptosis [103,104,105]. In line with this, expression of either IDO or IDO-derived catabolite, kynurenine, can exert immunosuppression.

Arginine has also been found to engage in the metabolic profile of immune cells [106]. Arginine is a key player in regulating T cell proliferation, differentiation, and survival [107]. Elevated levels of arginine induce a shift from glycolysis to OXPHOS in activated T cells to generate memory T cells, and enhance the capacity of T cells to eradicate tumors [107]. Arginine is also crucial in NK cells, as low concentrations of arginine impair proliferation as well as IFN-γ production in NK cells [108]. The enzyme ARG1, expressed by immunosuppressive cells such as TAMs and MDSCs can deplete arginine [109,110] by converting it into urea and ornithine, thus limiting the arginine available for T cell activation and anti-cancer activities [111]. Interestingly, nitric oxide synthase (NOS), an enzyme catalyzing the production of nitric oxide (NO) from arginine, is also considered to contribute to tumorigenesis [112,113]. A recent study has shown that NOS activity increases as cancer progresses, while a decrease in NOS is observed after chemotherapy [114]. It is well known that TGF-β takes part in regulating cellular proliferation and differentiation, however, its aberrant expression is often observed in TME [115]. Interestingly, after stimulation of TGF-β, the expression of ARG1 and IDO-1 is upregulated in DCs, and the activation of IDO-1 signaling is dependent on prior expression of ARG1 [97]. The dual expression of ARG1 and IDO-1 leads DCs toward a more immunosuppressive state. In line with this, the expression of ARG1 in the TME may limit the availability of arginine, which can inhibit the anti-tumor response of NK cells and T cells.

Glutamine is another element necessary for cellular proliferation and differentiation [116], and can promote proliferation in cancer cells [117,118]. Glutaminase (GLS) and glutamine synthetase (GS) are the two major enzymes involved in glutamine metabolism. GLS can regulate different subsets of T cells differently. Deficiency in GLS can deregulate T cell initial activation, proliferation and differentiation of Th17 cells, but it can also increase T-box expressed in T cells (Tbet) to promote differentiation and effector functions of CD4^+^ T cells and CD8^+^ T cells [119]. This distinct regulation of T cell by GLS may associated with T cell-mediated anti-tumor responses. It has been reported that cancer cells are induced to express GLS, converting glutamine into glutamate to fuel their rapid proliferation, resulting in invasion and metastasis in hypoxic conditions [120]. A recent study demonstrated that cancer cells could secrete exosomes to activate the glutamine and glutamate axis in CAFs, which indirectly supported the survival and proliferation of cancer cells [121]. GS also promotes Foxp3 expression in T cells as well as regulatory features in T_regs_ [122]. In addition, GS is a key regulator in macrophage polarization; significant expression of GS protein drives macrophages toward a M2-like phenotype [123], and high levels of GLS are found in M2 macrophages to sustain immunosuppressive phenotype [124]. M2-like TAMs are associated with a protumoral phenotype, however, administration of GS inhibitor causes a shift toward a M1-like phenotype [123]. In line with this, the glutamine and glutamate axis can reinforce immunosuppressive activities in immune cells, but also impede effector T cell functions, which in turn helps cancer cells to escape from immune surveillance.

#### 2.6.3. Lipid Metabolism

FAS and fatty acid oxidation (FAO) have both been reported to play central roles in lipid metabolism and the regulation of immune cells [125,126]. Lipid metabolism plays a role in the activation of both M1 and M2 macrophages. While fatty acid synthase (FASN) is a key enzyme for fatty acid biosynthesis, and plays an essential role in the induction of M1 macrophages [127], M2 macrophages mainly depend on FAO by oxidizing fatty acids to fuel OXPHOS [127]. Interestingly, M2 macrophages uptake triacylglycerol substrates via the scavenger receptor CD36 [128]. Triacylglycerol substrates undergo lipolysis by lysosomal acid lipase to support the elevated OXPHOS necessary for activation of M2 macrophages [128]. Recently, it has become clear that TAMs accumulate lipids through CD36 and serve as a source of FAO used for differentiation and tumor promotion [129].

Lipid metabolism has a distinct difference in subsets of T cells. FAS is harnessed to support effector T cell proliferation and differentiation [130], while the development of CD8^+^ memory T cells is depended on FAO [131]. Additionally, T_regs_ meet their energy demand primarily by FAO [132], and CD36 is also reported to be upregulated in intratumoral T_regs_ and to control their immunosuppressive functions [133]. Recently, a study reported that inhibition of CD8^+^ T cells can result from activation of STAT3 signaling, which can enhance FAO and promote obesity-associated breast cancer progression [134]. The study also showed that PD-1 ligation induces STAT3 signaling, enhancing FAO in CD8^+^ T cells, while inhibiting glycolysis and effector functions [134]. In line with this, lipid metabolism plays a critical role in regulating T cells. It may promote T_regs_ to generate an immunosuppressive TME, while dampening the capacity of CD8^+^ T cells to eradicate cancer cells.

Interestingly, a colorectal cancer (CRC) cell model showed that CAFs undergo a lipid metabolic reprogramming that leads them to accumulate more fatty acids and phospholipids [135]. The key enzyme FASN is significantly elevated in CAFs, releasing lipid metabolites that promote migration of CRC cells [135]. This CAF-induced CRC cell migration can be blocked by knocking down FASN in CAFs in vitro or by impeding fatty acid uptake by CRC cells using a CD36 monoclonal antibody in vivo [135].

A recent study showed that melanoma-derived Wnt5 can trigger β-catenin signaling in DCs, inducing the activation of peroxisome proliferator-activated receptor (PPAR) [136]. This Wnt5 signaling enhances FAO in DCs, but also increases IDO activity, which in turn promotes T_regs_ [136]. Additionally, the enhanced FAO suppresses the expression of proinflammatory cytokines IL-6 and IL-12 in DCs [136]. Together, the enhanced FAO leads DCs toward a more immunosuppressive state. Interestingly, prostaglandin E_2_ (PGE_2_) has been shown to be a crucial mediator in immune responses [137,138]. PGE_2_ upregulates IL-10 production in DCs [139], while downregulating MHC-II expression [140]. The decreased expression of MHC-II could impede antigen presentation by DCs and attenuate T cell-mediated immune responses.

## 3. FDA-Approved Immune Checkpoint Inhibitors and Metabolic Interventions

### 3.1. Immune Checkpoint Proteins in Neoplastic Development

Immune checkpoint proteins are mediators of the immune system, and are mainly two types of signals: co-stimulatory signals and co-inhibitory signals. These immune checkpoints are crucial for balancing self-tolerance and autoimmunity and work by sending signals to regulate immune cells [141]. Cancer cells, however, can utilize this regulatory mechanism to escape from immune surveillance. During the activation of T cells, co-inhibitory CTLA-4 is significantly upregulated and impedes T-cell receptor (TCR) signaling by competing with the co-stimulatory receptor CD28 for B7 ligands B7-1 (CD80) and B7-2 (CD86) that expressed by antigen presenting cells (APCs) [5]. It has been reported that T_regs_ express high levels of CTLA-4, and that these high-expressing CTLA-4 T_regs_ can be activated by binding to B7 ligands on APCs to exert immunosuppression [142], and also limit the availability of B7 ligands that are necessary for T cell activation. PD-1 is another important co-inhibitory checkpoint for balancing immune responses to chronic pathogens and cancer cells [143]. Upon activation, PD-1 expression in T cells is significantly upregulated and delivers inhibitory signals via binding to PD-1 ligands (PD-L1 and PD-L2) expressed by APCs or cancer cells [143], leading to dampened immune responses. It has become clearer that several neoplasms evade the immune surveillance by upregulating PD-L1 expressions that can bind to PD-1 expressed by T cells, contributing to T cell exhaustion [144]. The activation of PD-1 signaling in T cells may regulate their cytokines production such as IFN-γ, TNF-α, and IL-2, and also proliferation and cellular differentiation [145,146,147]. By utilizing this negative regulating pathway, cancer cells are able to survive and proliferate to sustain the neoplastic formation.

### 3.2. Immune Checkpoint Inhibitors

Since the discovery of immune checkpoint proteins, immune checkpoint inhibitors have revolutionized the approach to cancer treatment. Antibodies that block the PD-1 and PD-L1 axis or CTLA-4 have been developed and are able to produce durable clinical responses and prolong overall survival in cancer patients [148,149]. There are currently 7 immune checkpoint inhibitors approved by the FDA, including the CTLA-4 inhibitor ipilimumab; PD-1 inhibitors nivolumab, pembrolizumab, and cemiplimab; and PD-L1 inhibitors avelumab, durvalumab, and atezolizumab [1]. Patients with certain specific types of tumors may have durable clinical responses [2,3,4], however, the majority of clinical responses to ICIs remain unsatisfactory since some proportion of patients who received ICIs might have developed resistance to checkpoint therapy. One possible explanation is that cancer cells have been shown to disable antigen presentation naturally or induced by therapeutic strategies with the robust T cell immune surveillance, enabling cancer cells to evade from immuno-recognition [150,151,152]. Gene mutation in antigen-presenting protein beta-2-microglobulin (B2M) leads to the loss of MHC I presentation in cancer cells [150,152], contributing to escape from CD8^+^ T cell immune surveillance. Another possible explanation is that cancer cells exert genetic mutation in IFN-γ related signaling pathways Janus kinase 1 (JAK1) or Janus kinase 2 (JAK2), which make cancer cells less susceptible to T cell-mediated IFN-γ tumor suppression [150,153]. Another current possible explanation is the deregulation of immune-metabolism. Cancer cells and immunosuppressive cells in the TME can secrete a variety of cytokines or metabolites that may directly or indirectly impede anti-cancer immunity via altering their metabolic profiles [22,154,155]. It is promising that recent studies have identified several additional immune checkpoint targets including inhibitory pathway targets LAG-3 (lymphocyte activating gene-3), TIM-3 (T-cell immunoglobulin and mucin domain-3), TIGIT (T-cell immunoglobulin and ITIM domain), and VISTA (V-domain Ig-containing suppressor of T cell activation); and stimulatory pathway targets OX40 (CD134), ICOS (inducible T-cell co-stimulator), and GITR (glucocorticoid-induced tumor necrosis factor receptor-related protein) [156,157]. These novel targets may possibly lead to improved clinical outcomes with the use of immune checkpoint therapy, however, these novel targets will not be further discussed since this review mainly focus on the current FDA-approved ICIs.

### 3.3. Metabolic Interventions Combined with Immune Checkpoint Inhibitors

The application of ICIs that block the PD-1 and PD-L1 axis or CTLA-4 has yielded remarkable clinical responses for a subset of patients. However, some patients do not respond to this immunotherapy, which may be the result of either primary (de-novo) resistance or acquired resistance [154]. Recently, a growing number of studies have shed light on acquired resistance that may be a result of deregulation of immuno-metabolism [22,154,155]. The efficacy of ICI monotherapy may be limited by the immunosuppressive TME. Therefore, several ongoing clinical trials have emphasized targeting metabolic circuits in combination with ICIs to enhance anti-tumor responses.

An ongoing phase III clinical trial in head and neck squamous cell carcinoma (NCT03358472) has demonstrated that treatment with combination IDO-1 inhibitor epacadostat and anti-PD-1 antibody pembrolizumab resulted in a lower mortality rate than treatment with pembrolizumab alone (17.14 vs. 21.05%). Combination epacadostat and pembrolizumab also resulted in a lower rate of serious adverse events compared with pembrolizumab alone (35.29 vs. 42.11%). Similarly, in a phase II complete trial (NCT03322540) the same combination resulted in a lower mortality rate compared with pembrolizumab alone (17.33 vs. 22.08%). However, a completed phase III trial in unresectable or metastatic melanoma found that combination pembrolizumab and epacadostat failed to yield better clinical outcomes compared to pembrolizumab alone, as they observed no significant differences in progression-free survival or overall survival [158].

Interestingly, a phase I clinical trial in renal cell carcinoma patients (NCT02655822) demonstrated that administration of A_2A_R antagonist combined with atezolizumab provided positive clinical outcomes. More than 72% of patients in this trial were resistant or refractory to anti-PD-1 and PD-L1 therapy, and the majority of patients had PD-L1-negative tumors, which makes monotherapy with anti-PD-1 and PD-L1 unlikely to provide significant benefit [159]. This study showed that the combination of anti-PD-L1 antibody atezolizumab with the A_2A_R antagonist ciforadenant increased recruitment of cytotoxic T cells to tumor regions, and increased the diversity of T-cell receptors, which together prolonged overall survival of patients [159].

An ongoing phase I/II trial (NCT02903914) is targeting the arginine pathway with arginase inhibitor CB-1158 alone or in combination with anti-PD-1 antibody pembrolizumab. Since arginine is required for the activation and proliferation of T cells, treatment with arginase inhibitor is a potential strategy to provide bioavailable arginine for T cells. The preliminary results show that >90% of arginase is inhibited and arginine levels increase up to 4-fold [160]. The increased arginine levels are able to trigger immune responses and might synergize with ICIs. Several other trials are also testing the efficacy of various metabolic interventions in combination with ICIs (Table 1).

## 4. Targeting Metabolic Pathways in Combination with FDA-Approved ICIs and Its Underlying Mechanisms

**Figure 2:**
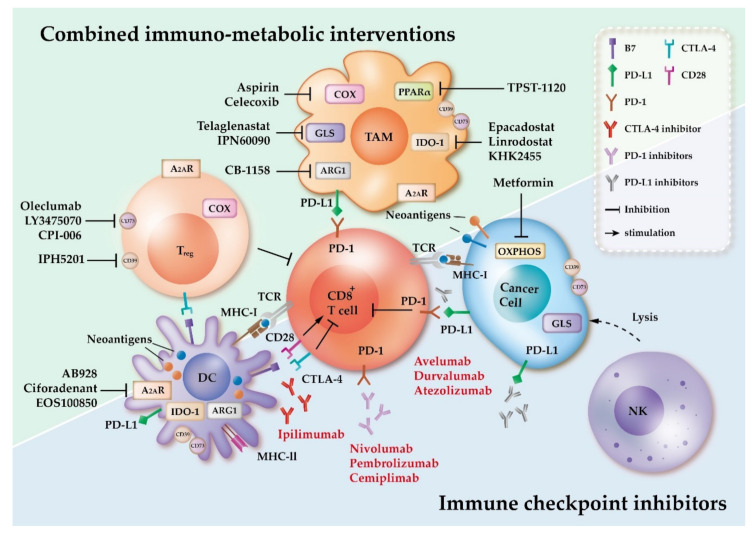
Targeting immuno-metabolic pathways to enhance the efficacy of immune checkpoint inhibitors. IDO-1, indoleamine 2,3-dioxygenase 1; COX, cyclooxygenase; ARG1, arginase 1; PPARα, proliferator-activated receptor α; A_2A_R, adenosine A_2A_ receptor; B7, B7-1 (CD80) and B7-2 (CD86) (not shown in Figure 2); PD-1, programmed cell death-1; PD-L1, programmed death-ligand 1; CTLA-4, cytotoxic T-lymphocyte-associated protein 4; MHC-I, major histocompatibility complex class I; MHC-II, major histocompatibility complex class II; TCR, T cell receptor; GLS, glutaminase; OXPHOS, oxidative phosphorylation; T_reg_, regulatory T cell; DC, dendritic cell; NK, natural killer cell; TAM, tumor associated macrophage.

Under harsh conditions of the TME nutrients are limited and cancer cells and immunosuppressive cells secrete cytokines and metabolites that can modulate the metabolic and functional activities of immune cells. The immunosuppressive TME imposes metabolic stresses on immune cells, dampening their capacity to eradicate cancer cells [161]. To date, only a small proportion of patients have shown durable responses to ICI therapy, which may partly result from the dysregulation of immune-metabolism [154]. In order to improve the response rate to ICIs, potential therapies aim to block the suppressive signals from immunosuppressive cells or to reactivate immune cells so that they regain anti-tumor functions [161]. Below, we discuss metabolic pathways that have been targeted in combination with FDA-approved ICIs, and propose potential underlying mechanisms in cellular metabolic rewiring. We attempt to provide a better understanding of the metabolic targets that may have synergistic effects with ICIs (Figure 2).

### 4.1. Targeting the Arginine Pathway

The metabolism of arginine plays a crucial role in the activation of T cells and regulates immune responses [97,106]. Arginase inhibitors may prevent arginine from degradation via inhibiting ARG1 that expressed mainly by DCs, TAMs, and MDSCs, leaving adequate amounts of arginine for T cell activation. It has recently been shown that CB-1158 (arginase inhibitor) can rescue the suppressed proliferation of T cells mediated by myeloid cells in vitro [162], and can increase the number of tumor-infiltrating CD8^+^ T cells and NK cells, reducing tumor growth, in vivo [162]. CB-1158 is currently under investigation in combination with pembrolizumab for the treatment of solid tumors (NCT02903914), and a possible underlying mechanism is that bioavailable arginine is scarce in the TME, limiting the efficacy of ICIs. With the use of CB-1158, it may provide bioavailable arginine for T cell activation, and the combination use of pembrolizumab can block PD-1 and PD-L1 ligation, thus abrogating inhibitory signals, which together may enhance T cell-mediated anti-cancer activities. Additionally, the inhibition of NOS may block the immunosuppressive activities in MDSCs and enhances anti-tumoral effects [163], indicating a NOS inhibitor may impede the production of NO in MDSCs, and might enhance the efficacy of ICIs since T cell is less suppressed and can exert effector functions. Currently, a NOS inhibitor, NG-Monomethyl-L-Arginine (L-NMMA), in combination with pembrolizumab, is being explored for the treatment of triple-negative breast cancer (NCT04095689).

### 4.2. Targeting the Tryptophan Pathway

Tryptophan can be metabolized into kynurenine by the enzyme IDO, and correlates with immunosuppressive properties [91,92,93]. A study has demonstrated that silencing IDO in DCs by small interfering RNA (siRNA) increases T cell proliferation and CD8^+^ T cell activity, while decreasing the number of T_regs_ [164]. The immunosuppressive effects on T cells exerted by TAMs are blocked via pre-treating IDO inhibitors to TAMs [165]. Importantly, a recent study demonstrated that with the administration of IDO inhibitors in combination with ICIs, CD8^+^ T cells were able to produce IL-2, TNF-α, and IFN-γ significantly comparing to either ICIs or IDO inhibitor alone, indicating that the combination treatment of IDO inhibitor with ICIs may enhance polyfunctional T cells in the TME [166]. The combination use of IDO inhibitor with ICIs significantly attenuates tumor growth, mainly via reactivation of T cells, while also increasing IL-2 production and proliferation of CD8^+^ T cells [166]. Therefore, targeting tryptophan metabolism with IDO inhibitors may reduce suppressive signals from immunosuppressive cells, and restore T cell-mediated anti-tumor responses. Several ongoing trials are targeting tryptophan metabolism with ICIs for the treatment of certain cancers (Table 1).

### 4.3. Targeting the Cyclooxygenase and PGE_2_ Pathway

Arachidonic acid is oxidized into PGE_2_ by the cyclooxygenase (COX) enzymes, and can regulate both innate and adaptive immunity [137]. COX-2 has been found to be overexpressed in many types of cancers, and to correlate with the promotion of carcinogenesis [167]. It has been reported that high levels of COX-2 in TAMs is crucial in maintaining M2-like phenotype, and can induce COX-2 expression in cancer cells, which promotes proliferation and survival of cancer cells [168]. In addition, cancer-derived PGE_2_ can suppress the cytotoxicity and differentiation of NK cells [169], while also dampening NK-mediated recruitment of DCs [170]. Moreover, the presence of COX-2 in TME can increase the accumulation of T_regs_ [171], while T_regs_ can also express COX-2 and produce PGE_2_, which is required to induce Foxp3 expression in T_regs_ and further inhibit T cell-mediated responses [172]. Together, the COX-2 and PGE_2_ pathway can promote immunosuppressive signals in TME and boost tumor promotion. Thus, inhibition of the COX-2 and PGE_2_ axis is a potential therapeutic target (Figure 2).

COX enzyme inhibitors such as aspirin and celecoxib may serve as anti-inflammatory agents that interfere with COX-2 mediated inflammatory responses in cancer [173]. It has been reported that administration of selective COX-2 inhibitor, celecoxib, can suppress macrophage infiltration and tumorigenesis [174]. They also found out that administration of celecoxib significantly decrease **CXC** chemokine ligand 2 (CXCL2) and N-cadherin expression in gastric tumor mouse model [174], indicating the use of COX enzyme inhibitor may suppress tumor engraftment and metastasis. Additionally, it has been shown that COX enzyme inhibitors in combination with anti-PD-1 treatment exhibit a synergistic effect in reducing tumor growth compared with either COX enzyme inhibitors or anti-PD-1 antibodies alone [175]. Taken together, these findings suggest that the combination of COX enzyme inhibitors with ICIs could be a therapeutic strategy for cancer treatment. Currently, these combination therapies are under examination in several clinical trials (Table 1).

### 4.4. Targeting the Glutamine and Glutamate Pathway

Glutamine can be converted to glutamate by GLS, and can be harnessed by cancer cells to promote proliferation [117,118]. A recent study in a tumor-bearing mouse model showed that restriction of glutamine could efficiently eradicate tumors and prolong survival via increasing CD8^+^ T cell activity [176]. GLS inhibitors including BPTES (bis-2-(5-phenylacetamido-1,3,4-thiadiazol-2-yl)ethyl sulfide) and CB-839 have also been demonstrated to suppress tumor growth in liver and breast cancer mouse models [177,178]. Additionally, GLS inhibitor BPTES can impeded M2-related gene expression in IL-4-treated macrophages [124]. Another study showed that combination treatment of GLS inhibitors with ICIs significantly decreased tumor volume in an ICI-resistant-tumor mouse model, and the possible underlying mechanism may via enhancing CD8^+^ T cell activities and by decreasing ARG1-expressing myeloid cells [179]. This study suggests that GLS inhibitors can enhance the efficacy of ICIs, by reinforcing the cytotoxic activities of CD8^+^ T cells and reshaping the immunosuppressive TME. There are several ongoing clinical trials that are investigating GLS inhibitors combined with ICIs for the treatment of solid tumors or advanced tumors (Table 1).

### 4.5. Targeting the Adenosine Pathway

Ectonucleotidases CD39 and CD73 both engage in the adenosinergic pathway [180], and have been used as prognostic biomarkers in several types of cancers [181]. T_regs_, macrophages and DCs are found to co-express CD39 and CD73 [182,183], enabling them to generate adenosine. It has been reported that extracellular levels of adenosine in the tumor environment are able to suppress anti-tumor immune responses [184]. In addition, the generation of adenosine via CD39 and CD73 in cancer cells may contribute to the recruitment of TAMs, and since TAMs also express elevated CD39 and CD73 this results in amplifying the immunosuppressive adenosine level [185]. The activation of A_2A_R or A_2B_R may also result in immunosuppression [186]. Together, the adenosinergic pathway appears to regulate immune responses and create an immunosuppressive TME [181,184,185,186]. Therefore, inhibiting the adenosine pathway might result in a less immunosuppressive TME and may boost immune responses (Figure 2).

It was recently shown that inhibition of CD39 enhances the activity of NK cells and inhibits cancer cell metastasis [187]. Likewise, co-inhibition of CD73 and A_2A_R in leukocytes was shown to limit tumor initiation, growth, and metastasis in a tumor-bearing mouse model [188]. Another study has demonstrated potential therapeutic effects of combining CD39 inhibitors with ICIs, showing a significant decrease in tumor size and prolonged survival in a melanoma mouse model [189]. Similarly, ICI treatment showed a positive therapeutic response in a CD39-deficient tumor-bearing mouse model [190]. Recently, the A_2A_R antagonist, ciforadenant (CPI-444), was administrated in combination with ICIs to different types of tumor mouse models [191]. The combination treatment of ciforadenant with anti-PD-1 improved tumor regression compared to treatment with anti-PD-1 alone [191], and one possible mechanism may due to a decrease in PD-1 expression in A_2A_R antagonist treated CD8^+^ T cells since decreasing PD-1 expression in CD8^+^ T cells may lower the threshold and increase the sensitivity of anti-PD-1 therapy [192]. A clinical trial combining ciforadenant with atezolizumab is now ongoing (NCT02655822). Many other adenosine-associated inhibitors combined with ICIs are also being tested (Table 1).

### 4.6. Targeting Glucose Metabolism

Glucose is largely consumed by cancer cells and may impede T cell-mediated anti-cancer activities [20]. Recently, researchers have discovered some types of cancers may upregulate OXPHOS to promote metastasis and progression [89,90]. Metformin, a widely prescribed drug for type II diabetes, is also considered as an OXPHOS inhibitor [193] that can stimulate the AMP activated protein kinase (AMPK) signaling pathway while inhibiting mTOR, and consequently exhibiting anti-tumorigenic effects [194,195]. Metformin inhibited tumor growth in a colorectal cancer patient-derived xenograft mouse model [196], via an increase in apoptotic Bax levels and a decrease in anti-apoptotic Bcl-2 levels [197]. Interestingly, metformin also inhibits oxygen consumption by cancer cells, thereby increasing the availability of oxygen in cancer regions and reducing intratumoral hypoxia [198]. The capacity of CD8^+^ T cells to secrete effector cytokines is enhanced by administration of metformin with anti-PD-1 compared with either metformin or anti-PD-1 alone [198]. Therefore, metformin combined with ICIs may have synergistic effects in stimulating T cell functions and eradicating cancer cells. Several clinical trials are now testing metformin in combination with ICIs to see if this can yield better clinical outcomes than monotherapy (Table 1).

### 4.7. Targeting Lipid Metabolism

FAS and FAO are both important factors that engage in the lipid metabolism and may exert regulation of immune cells [125,126]. Inhibition of FASN has been observed to upregulate expression of CD36, which can compensate for the anti-tumor effects of FASN inhibition [199]. Therefore, inhibiting CD36 should improve the efficacy of FASN-targeted therapy. A recent study shows that genetic deletion of CD36 in T_regs_ can shift their metabolic profile from OXPHOS to glycolysis, and can induce apoptosis of intratumoral T_regs_ [133]. They also demonstrate that in a melanoma-bearing mouse model combination treatment of anti-CD36 antibody combined with anti-PD-1 shows a stronger capability to restrict cancer growth compared with either anti-CD36 or anti-PD-1 alone [133]. Administration of anti-CD36 with ICIs has not yet been studied in clinical trials. Importantly, the ligand-activated nuclear transcription factor PPARα has been shown to regulate lipid metabolism and FAO [200], thus, a selective PPARα antagonist may be effective in shifting intracellular metabolism from FAO to glycolysis [201], skewing FAO-dependent M2 macrophages toward an M1 phenotype [127]. The PPARα antagonist TPST-1120 in combination with nivolumab is being studied for the treatment of advanced cancers (NCT03829436).

## 5. Conclusions

The application of ICIs has revolutionized clinical treatments for cancer patients, however only a subset of recipients has shown durable responses to ICIs, and some patients develop resistance. The lack of response to ICI treatment may be the result of dysfunction of cellular metabolism, as more and more researches have demonstrated correlation between cellular metabolism and resistance. As shown in Figure 1, immune cells are forced to undergo metabolic reprogramming due to environmental stresses. These metabolic alterations may further create an immunosuppressive TME, but could also impede anti-tumor responses. In order to block cancer-induced metabolic reprogramming, several ongoing clinical trials are targeting different metabolic pathways in combination with ICIs (Table 1). As more findings suggest that metabolic intervention could be an effective strategy for improving the efficacy of ICIs, it has become increasingly important to better understand cellular metabolism in the TME in order to overcome ICI resistance. In this review, we discuss the metabolic features of immune cells within the TME (Figure 1), and list several ongoing clinical trials of ICIs in combination with metabolic interventions (Table 1). We also propose possible underlying mechanisms of metabolic inhibitors to reprogram the immunosuppressive TME (Figure 2). With the map of cellular metabolic interactions in immune cells and cancer cells, we have attempted to provide a better understanding of the metabolic crosstalk in the TME, with the hope that this will aid in overcoming ICI resistance.

## Figures and Tables

**Table 1:** Ongoing clinical trials targeting metabolic circuits in combination with immune checkpoint inhibitors.

Metabolic Targets	Immune Checkpoint Inhibitors	Cancer Types	Phase	Status	Clinical Trial Identifier
*Arginine pathway inhibitors*					
**L-NMMA** **(NO synthase inhibitor)**	Pembrolizumab	TNBC	II	Not yet recruiting	NCT04095689
**CB-1158** **(Arginase inhibitor)**	Pembrolizumab	Solid tumors	I/II	Active, not recruiting	NCT02903914
*IDO inhibitors*					
**Epacadostat** **(INCB024360;** **IDO-1 inhibitor)**	Pembrolizumab	HNSCC	III	Active, not recruiting	NCT03358472
	Pembrolizumab	RCC	III	Active, not recruiting	NCT03260894
	Pembrolizumab	GIST	II	Active, not recruiting	NCT03291054
	Pembrolizumab	MIBC	II	Not yet recruiting	NCT03832673
	Pembrolizumab	Thymic cancer	II	Active, not recruiting	NCT02364076
	Pembrolizumab	Metastatic pancreatic cancer	II	Recruiting	NCT03006302
	Pembrolizumab	Sarcoma	II	Active, not recruiting	NCT03414229
	Ipilimumab + Nivolumab	Solid tumors	I/II	Active, not recruiting	NCT03347123
**Linrodostat** **(BMS-986205;** **IDO-1 inhibitor)**	Nivolumab	Melanoma	III	Active, not recruiting	NCT03329846
	Nivolumab	Endometrial cancer	II	Recruiting	NCT04106414
	Nivolumab	HNSCC	II	Recruiting	NCT03854032
	Nivolumab	HCC	I/II	Recruiting	NCT03695250
	Nivolumab	NSCLC	I/II	Recruiting	NCT02658890
	Nivolumab	Solid tumors	I/II	Active, not recruiting	NCT03792750
**PD-L1/IDO peptide vaccine**	Nivolumab	Melanoma	I/II	Recruiting	NCT03047928
**KHK2455** **(IDO-1 inhibitor)**	Avelumab	Bladder cancer	I	Recruiting	NCT03915405
*Inhibitors of COX enzymes*					
**Aspirin** **(COX-1 and COX-2 inhibitor)**	Atezolizumab	Ovarian cancer	II	Active, not recruiting	NCT02659384
	Avelumab	TNBC	II	Not yet recruiting	NCT04188119
	Ipilimumab + Pembrolizumab	Melanoma	II	Active, not recruiting	NCT03396952
	Pembrolizumab	CRC	II	Recruiting	NCT03638297
	Pembrolizumab	Cervical/Uterine cancer	II	Recruiting	NCT03192059
**Celecoxib** **(COX-2 inhibitor)**	Pembrolizumab	Brain metastasis from TNBC or HER2^+^ breast cancer	II	Not yet recruiting	NCT04348747
	Nivolumab	Solid tumors	II	Not yet recruiting	NCT03864575
**Grapiprant** **(EP4 antagonist)**	Pembrolizumab	NSCLC	I/II	Recruiting	NCT03696212
	Pembrolizumab	Microsatellite stable CRC	I	Recruiting	NCT03658772
*Glutamine and glutamate pathway inhibitors*					
**Telaglenastat** **(CB-839;** **glutaminase inhibitor)**	Pembrolizumab	NSCLC	II	Recruiting	NCT04265534
	Nivolumab	Melanoma or NSCLC	I/II	Active, not recruiting	NCT02771626
**DRP-104** **(glutamine antagonist)**	Atezolizumab	Solid tumors	I/II	Recruiting	NCT04471415
**IPN60090** **(glutaminase inhibitor)**	Pembrolizumab	Solid tumors	I	Recruiting	NCT03894540
*Adenosine pathway inhibitors*					
**Oleclumab** **(MEDI9447;** **anti-CD73 antibody)**	Durvalumab	Luminal B breast cancer	II	Active, not recruiting	NCT03875573
	Durvalumab	TNBC	I/II	Recruiting	NCT03616886
**AB928** **(A_2A_R and A_2B_R antagonist)**	Atezolizumab	CRC	I/II	Recruiting	NCT03555149
**Ciforadenant** **(CPI-444;** **A_2A_R antagonist)**	Atezolizumab	RCC	I	Recruiting	NCT02655822
**AZD4635** **(A_2A_R antagonist)**	Durvalumab	NSCLC or CRC	I	Active, not recruiting	NCT02740985
**IPH5201** **(Anti-CD39 antibody)**	Durvalumab	Solid tumors	I	Recruiting	NCT04261075
**LY3475070** **(CD73 inhibitor)**	Pembrolizumab	Advanced cancers	I	Recruiting	NCT04148937
**CPI-006** **(Anti-CD73 antibody)**	Pembrolizumab	Advanced cancers	I	Recruiting	NCT03454451
**EOS100850** **(A_2A_R antagonist)**	Pembrolizumab	Solid tumors	I	Recruiting	NCT03873883
*Inhibitors of glucose metabolism*					
**Metformin** **(Multiple effects of glucose metabolism)**	Pembrolizumab	HNSCC	II	Recruiting	NCT04414540
	Nivolumab	NSCLC	II	Active, not recruiting	NCT03048500
	Durvalumab	HNSCC	I	Recruiting	NCT03618654
	Pembrolizumab	Melanoma	I	Recruiting	NCT03311308
*Inhibitors of lipid metabolism*					
**TPST-1120** **(PPAR** **α antagonist)**	Nivolumab	Advanced cancers	I	Recruiting	NCT03829436

TNBC, triple-negative breast cancer; HNSCC, head and neck squamous cell carcinoma; RCC, renal cell carcinoma; GIST, gastrointestinal stromal tumor; NSCLC, non-small cell lung cancer; MIBC, muscle-invasive bladder cancer; HCC, hepatocellular carcinoma; CRC, colorectal cancer.

## Data Availability

Data is contained within the article.
